# Nonconvulsive Status Epilepticus Caused by Cerebrospinal Fluid Dissemination of a Salivary Duct Carcinoma: A Case Report

**DOI:** 10.31662/jmaj.2021-0108

**Published:** 2021-12-28

**Authors:** Takahisa Kato, Junya Tsurukiri, Hidefumi Sano, Takeo Nagura, Mariko Moriya, Hiroki Suenaga, Kyosuke Matsunaga, Takeshi Kanemura, Yuki Ueta, Takao Arai

**Affiliations:** 1 Department of Emergency and Critical Care Medicine, Tokyo Medical University Hachioji Medical Center, Tokyo, Japan; 2 Department of Neurology, Tokyo Medical University Hachioji Medical Center, Tokyo, Japan

**Keywords:** epilepsy, nonconvulsive status epilepticus, malignant tumor, disturbance of consciousness, salivary duct carcinoma

## Abstract

Salivary duct carcinoma (SDC) is a rare and highly aggressive salivary gland tumor with rapid growth, distant metastasis, and a high recurrence rate. Moreover, the parotid gland is the most common site with a poor prognosis. A lower frequency of distance metastasis to the liver, skin, and brain has also been reported, although the lungs, bones, and lymph nodes are the most common sites of SDC metastasis. We report a case of nonconvulsive status epilepticus (NCSE) in a 73-year-old male comatose patient having SDC of the parotid gland with an unusual metastasis to the skin and brain diagnosed by frequent cerebrospinal fluid examinations. Meningeal carcinomatosis usually has a poor prognosis, and NCSE is a reversible cause of altered mentation. Clinicians should know the unique set of epilepsy etiologies in patients with malignant tumors.

## Introduction

Salivary duct carcinoma (SDC) is a rare salivary gland tumor that occurs in the ductal epithelium of the salivary gland, is often difficult to treat, and is associated with poor outcomes ^(1)^. Pulmonary and bone metastases are common, whereas skin and central nervous system (CNS) metastases are uncommon ^(2)^. We treated a patient presenting with disturbance of consciousness (DOC) caused by nonconvulsive status epilepticus (NCSE), who developed SDC and metastases to the meninges.

## Case Report

**Figure 1.**
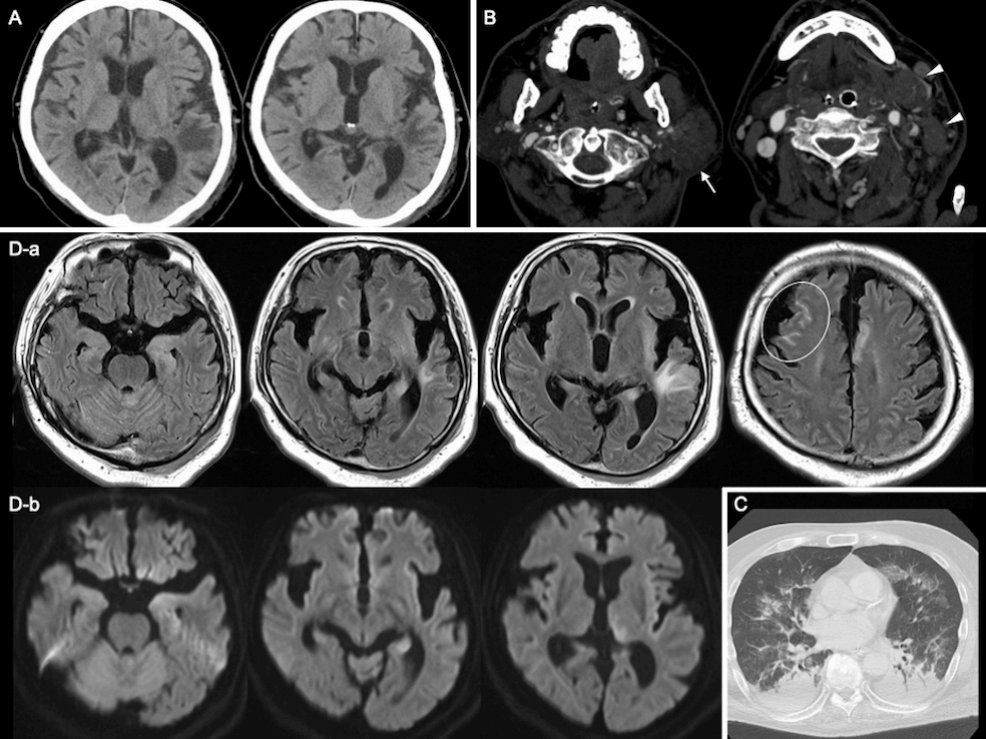
Images *Computed tomography (CT) images* A) Head CT image revealed a low-intensity area at the left temporal lobe. B) Cervical CT imaging showed lymphadenopathy from the left neck to the left axilla (arrow heads) and a continuous mass lesion in the left parotid glands (arrow). C) Chest CT imaging showed bilateral pleural effusion and passive atelectasis, and infiltrative shadows in the bilateral upper lobe, middle lobe, and lingular segment. *Magnetic resonance (MR) images* D-a) Fluid-attenuated inversion recovery imaging of the brain revealed a high intensity in the left hippocampus, insula, and cingula, and edematous change of the left temporal lobe. Hyperintensity in the subarachnoid space of the right frontal lobe suggesting meningeal carcinomatosis (circle). D-b) Diffusion-weighted imaging signal hyperintensity in the left pulvinar of the thalamus and hippocampus. These findings suggested NCSE.

A 73-year-old male was transferred to our emergency center because of DOC with no remarkable past medical history. His physical examination revealed the following: Glasgow Coma Scale (GCS) score, E1V1M1; pupil diameter, 2 mm; blood pressure, 106/78 mmHg; heart rate, 150 beats/min; respiratory rate, 28 breaths/min; and body temperature, 36.6°C. We observed muscle twitching in the upper limbs and face, and bedsores of the buttock, tendon reflexes were normal, and the Babinski sign was indifferent. We also observed a well-defined elastic solid mass (48 × 31 mm) in the left submandibular triangle and multiple infiltrative solid elastic red plaques/nodules in the left chest. His laboratory examinations are listed in Table 1. Computed tomography (CT) of the head revealed a low-density area in the left temporal lobe suspected to be edematous. Cervical CT imaging showed lymphadenopathy of the left neck and a continuous mass in the left parotid gland. Chest CT imaging showed pneumonia (Figure 1A, B and C).

**Table 1.** Laboratory Test and Cerebrospinal Fluid Examination on Arrival at Our Emergency Department.

*Variables*				
Laboratory tests			Reference range	
White blood cells	6,610	/µL	3,600–9,000	/µL
Red blood cells	501	/µL	387−525	/µL
Hemoglobin	15.9	g/dL	12.6–16.5	g/dL
Hematocrit	49	%	37.4−48.6	%
Platelet	14.6	/µL	13.8−30.9	/µL
Aspartate aminotransferase	108	U/L	30	U/L
Alanine aminotransferase	39	U/L	30	U/L
Creatine kinase	3077	U/L	60−287	U/L
Urea nitrogen	50.9	mg/dL	7−24	mg/dL
Creatinine	2.54	mg/dL	1.00	mg/dL
Sodium	146	mg/dL	135−147	mg/dL
Potassium	4.2	mg/dL	3.3−4.8	mg/dL
Chloride	105	mg/dL	98−108	mg/dL
Glucose	97	mg/dL	78−109	mg/dL
Ammonia	22	µg/dL	30−80	µg/dL
C reactive protein	38.59	mg/dL	0.3	mg/dL
Prothrombin-international normalized ratio	1.21		0.9−1.1	
Activated partial thromboplastin time	35.4	sec	27−40	sec
D-dimer	29.3	µg/dL	1.0	µg/dL
**Cerebrospinal fluid examination**				
Pressure	240	mmHg	50−80	mmHg
Quantitative protein	63	mg/dL	15−40	mmHg
Quantitative glucose	46	mg/dL	45–80	mg/dL
Cell count	2	/uL	< 5	/uL
Bacterial culture	negative			
Herpes smplex virus DNA	negative			
Varicella zoster virus DNA	negative			

DNA, deoxyribonucleic acid

**Figure 2.**
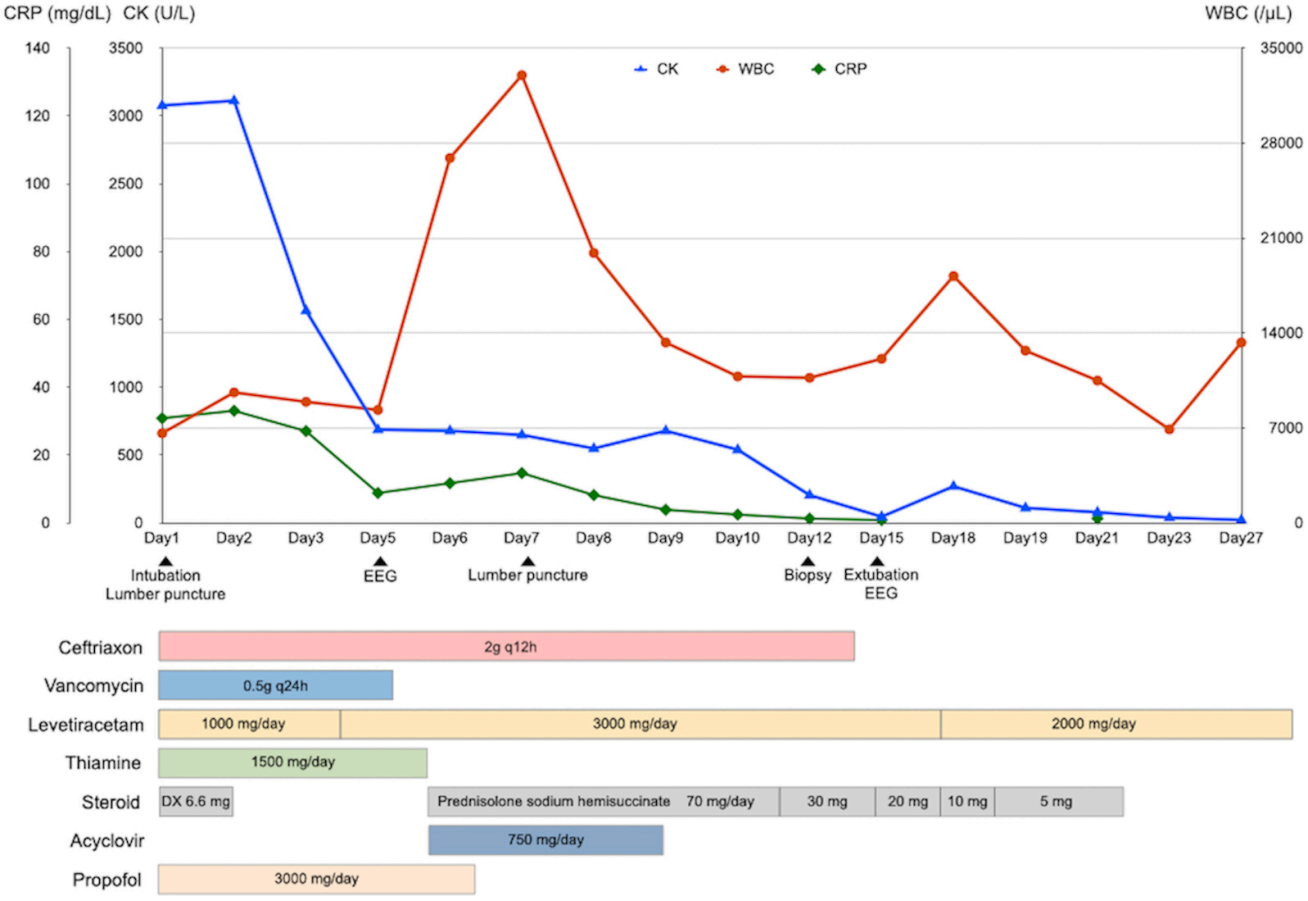
Critical care at the intensive care unit EEG, electroencephalogram; DX dexamethasone.

**Figure 3.**
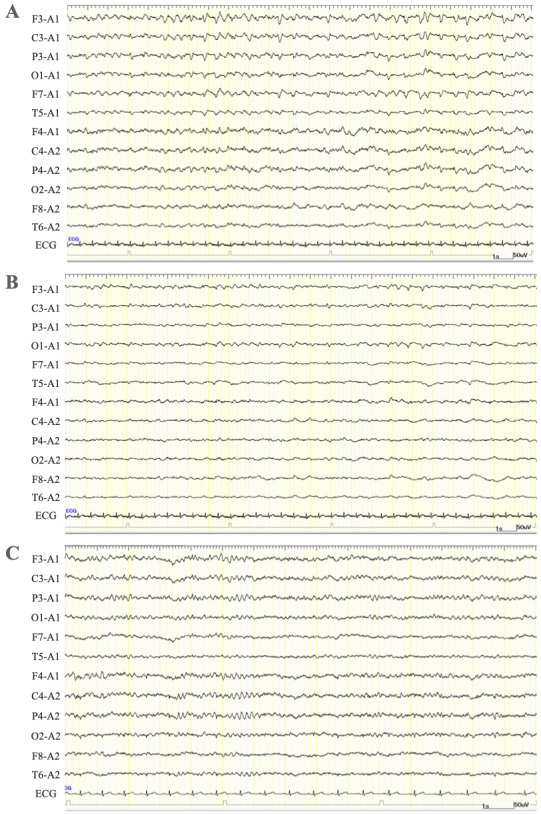
Electroencephalogram at the onset (A: the monopolar montage; B: the longitudinal bipolar montage) and after starting levetiracetam (C) A, B) Lateralized rhythmic delta activity (LRDA) in the left frontal parietal region began as low-voltage 3 Hz blunt waves and changed to triphasic morphology. After that, lateralized periodic discharges (LPDs) with triphasic morphology appeared. The frequency fluctuated between 1.5 and 2 per second. C) LRDA and LPDs disappeared, and alpha and theta waves at 7-9 Hz were irregular and unsteady.

**Figure 4.**
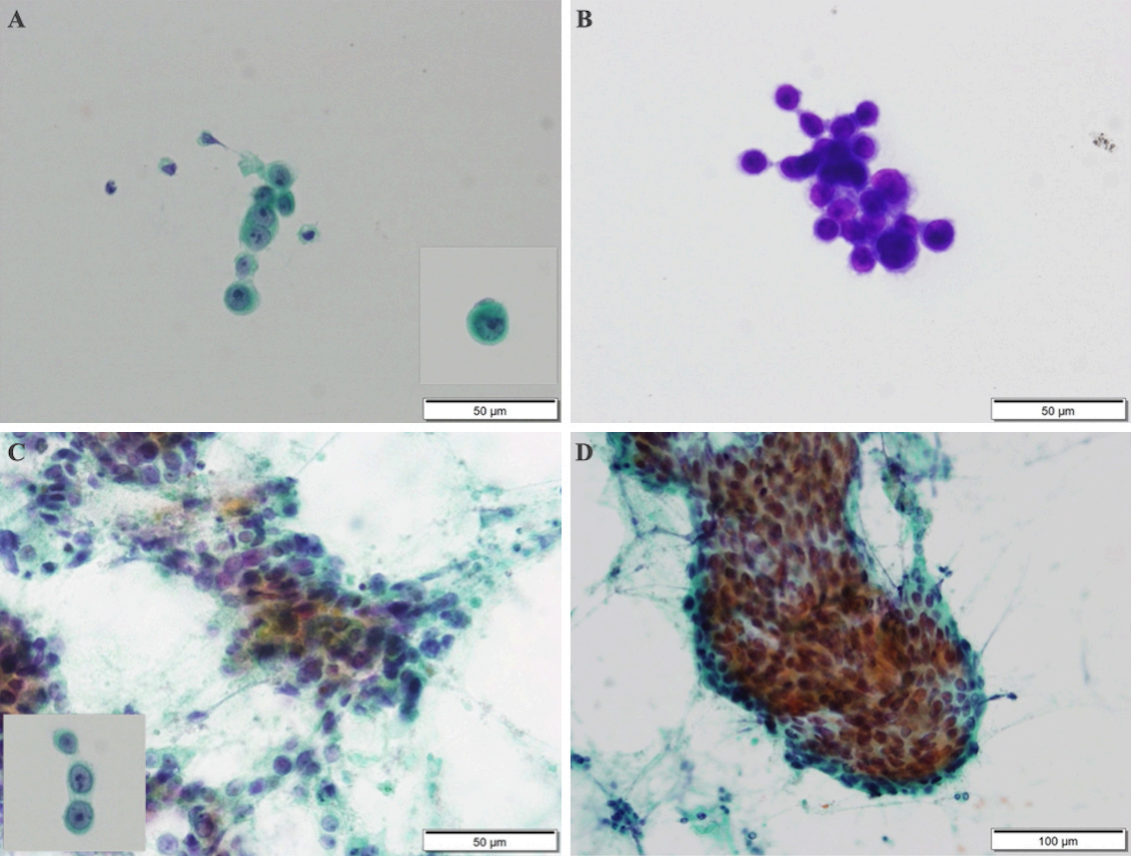
*Cytodiagnosis of the cerebrospinal fluid* (A) Papanicolaou stain. Solitary or clustered large lymphoid cells with conspicuous nucleoli were determined. (B) Periodic acid Schiff staining was positive, and adenocarcinoma was suspected. *Papanicolaou stain of the neck mass* (C) Colonization of the epidermal atypical cells was determined with a background of massive necrosis. Histopathological findings of the neck mass were similar to those of the cerebrospinal fluid. (D) Tubular formations were determined.

The patient was diagnosed with sepsis and required mechanical ventilation and critical care management under anesthesia. His clinical course is shown in Figure 2. NCSE, bacterial meningitis, or Wernicke’s encephalopathy was also suspected, and levetiracetam (1000 mg/day), vitamin B1, and intravenous steroids were initially administered. Magnetic resonance imaging of the brain revealed abnormal intensity in both cerebral hemispheres, suggesting NCSE or limbic encephalitis, and fluid-attenuated inversion recovery imaging revealed high intensity in the subarachnoid space, suggesting meningeal carcinomatosis (Figure 1D). Electroencephalography (EEG) showed lateralized rhythmic delta activity (LRDA) and lateralized periodic discharges (LPDs) with triphasic morphology predominantly in the left frontal parietal region fluctuating in frequency and morphology (Figure 3A, B) ^(3)^. CSF cytology showed large lymphocyte-like cells with prominent nucleoli, which was a Class V malignant tumor on day 7 (Figure 4A, B). His consciousness improved to GCS E4VtM5, and the patient was extubated. On day 12, a skin biopsy of the left chest skin eruption revealed metastatic skin cancer. Furthermore, the left cervical lymph node was biopsied and revealed a Class V SDC (Figure 4C, D). The clinical diagnosis of NCSE caused by meningeal carcinomatosis was posed, and a repeat EEG on day 15 revealed disappearance of LRDA (Figure 3C). The patient was discharged from the hospital to another hospital after 37 days.

## Discussion

SDC increases in neck masses or facial nerve paralysis are likely to become symptomatic ^(4)^. Metastases to distant organs occur in 40%-70% of adult patients, with pulmonary, bone, and lymph node metastases being the most common, whereas liver, skin, and CNS are less common ^(2)^. Although the standard treatment for SDC is radical surgery, the recurrence rate is high^(2), (5)^. Past studies reveal skin and brain metastases in one patient requiring long-term follow-up even after remission ^(6)^.

Meningeal carcinomatosis is a disease associated with poor prognoses, presenting with meningeal dissemination in which cancer cells float in the CSF and proliferate diffusely in the pia mater of the brain and subarachnoid space ^(7)^. In this patient, CSF tests conducted by several lumbar punctures for the differential diagnosis of DOC showed increased CSF pressure and quantitative protein, and cytology revealed meningeal carcinomatosis of the SDC. Furthermore, on the basis of the original EEG findings and a positive electroclinical response to antiepileptic therapy, we diagnosed NCSE due to meningeal carcinomatosis.

The etiologies of NCSE are poor compliance to medications, alcohol intoxication, infection, stroke, CNS tumors, or trauma ^(8)^. NCSE occurs in 6%-8% of patients with cancer, and it can be the presenting sign of a new brain disorder or metastasis ^(9)^. NCSE is a likely cause of altered mental status in patients with encephalopathy without organ failure. Therefore, early administration of antiepileptic drugs should be a priority ^(10)^.

In conclusion, NCSE can be a reversible cause of altered mentation, although meningeal carcinomatosis is associated with a poor prognosis. Clinicians should be aware of the unique set of NCSE etiologies in patients with rare malignant tumors, and once these are identified, consideration of a rapid multidisciplinary diagnostic approach is essential for making decisions regarding the appropriate treatment.

## Article Information

### Conflicts of Interest

None

### Acknowledgement

The authors would like to thank Dr. Hirano Hiroshi and Dr. Wakiya Midori (Pathology department of Tokyo Medical University Hachioji Medical Center) for their pathological diagnosis, and Enago (www.enago.jp) for the English language review.

### Author Contributions

Conceived and designed the experiments: KT, TJ

Contributed to interpretation of data: SH, MK, NT, MM, SH, KT

Approved the final version to be submitted: UY, AT

### Informed Consent

Written informed consent was obtained from the next of kin of the patient for publication of this case report and accompanying images.

## References

[1] Gilbert MR, Sharma A, Schmitt NC, et al. A 20-year review of 75 cases of salivary duct carcinoma. JAMA Otolaryngol Head Neck Surg. 2016;142(5):489-95. 26939990 10.1001/jamaoto.2015.3930 PMC5033043

[2] Otsuka K, Imanishi Y, Tada Y, et al. Clinical outcomes and prognostic factors for salivary duct carcinoma: a multi-institutional analysis of 141 patients. Ann Surg Oncol. 2016;23(6):2038-45. 26790669 10.1245/s10434-015-5082-2 PMC4858547

[3] Trinka E, Leitinger M. Which EEG patterns in coma are nonconvulsive status epilepticus? Epilepsy Behav. 2015;49:203-22. 26148985 10.1016/j.yebeh.2015.05.005

[4] Stodulski D, Mikaszewski B, Majewska H, et al. Parotid salivary duct carcinoma: a single institution’s 20-year experience. Eur Arch Otorhinolaryngol. 2019;276(7):2031-8. 31062093 10.1007/s00405-019-05454-0 PMC6581927

[5] Han MW, Roh JL, Choi SH, et al. Prognostic factors and outcome analysis of salivary duct carcinoma. Auris Nasus Larynx. 2015;42(6):472-7. 26028371 10.1016/j.anl.2015.04.005

[6] Sadeghi HM, Karimi A, Rahpeima A, et al. Salivary duct carcinoma with late distant brain and cutaneous metastasis: a case report. Iran J Pathol. 2020;15(3):521-5. 32754223 10.30699/ijp.2020.103326.2039 PMC7354077

[7] Lombardi G, Zustovich F, Farina P, et al. Neoplastic meningitis from solid tumors: new diagnostic and therapeutic approaches. Oncologist. 2011;16(8):1175-88. 21795431 10.1634/theoncologist.2011-0101 PMC3228160

[8] Grewal J, Grewal HK, Forman AD. Seizures and epilepsy in cancer: etiologies, evaluation, and management. Curr Oncol Rep. 2008;10(1):63-71. 18366962 10.1007/s11912-008-0010-2

[9] Wang N, Bertalan MS, Brastianos PK. Leptomeningeal metastasis from systemic cancer: review and update on management. Cancer. 2018;124(1):21-35. 29165794 10.1002/cncr.30911 PMC7418844

[10] Gutierrez C, Chen M, Feng L, et al. Non-convulsive seizures in the encephalopathic critically ill cancer patient does not necessarily portend a poor prognosis. J Intensive Care. 2019;7:62. 31890224 10.1186/s40560-019-0414-0 PMC6915900

